# Machine Learning-Based Prediction of Masaoka–Koga Stage and WHO Histological Risk Group in Thymic Epithelial Tumors Using Biomarker Combinations

**DOI:** 10.3390/diagnostics16132118

**Published:** 2026-07-07

**Authors:** Konstantinos Kitrou, Georgios Mandrakis, Georgios Tsirogiannis, Stamatios Theocharis, Constantinos Halkiopoulos, Yannis Stamatiou

**Affiliations:** 1 Department of Business Administration, University of Patras, 26504 Rio, Achaia, Greece; 2 First Department of Pathology, School of Medicine, National and Kapodistrian University of Athens, 75 Mikras Asias Street, 11527 Athens, Greece; stamtheo@med.uoa.gr; 3 Department of Food Science and Technology, University of Patras, 26504 Rio, Achaia, Greece; gtsirogianni@upatras.gr; 4 Department of Management Science and Technology, University of Patras, 26504 Patras, Greece; halkion@upatras.gr

**Keywords:** thymic epithelial tumors, Masaoka–Koga staging, WHO classification, machine learning, logistic regression, XGBoost, EphA6, YAP, TAZ, HDAC4

## Abstract

**Background:** Thymic epithelial tumors (TETs) are the most common primary neoplasms of the anterior mediastinum and present a dual classification challenge, namely anatomical staging according to the Masaoka–Koga system and histological risk stratification according to the World Health Organization (WHO) classification. Both tasks rely on expert pathological assessment and may be affected by interobserver variability. This study applied supervised machine learning (ML) to quantitative immunohistochemical (IHC) H-score profiles to predict Masaoka–Koga stage and WHO risk group in TETs. **Methods**: Logistic regression (LR) and XGBoost were applied to 19 biomarkers, including cellular localization, across two parallel analyses. Masaoka–Koga stage prediction was performed in 81 patients, including 59 early-stage and 22 advanced-stage cases, using the Synthetic Minority Oversampling Technique (SMOTE) across 100 train/test splits. WHO risk group prediction was performed in 89 patients, including 45 low-risk and 44 high-risk tumors, without oversampling. A cross-endpoint analysis applied the optimal Masaoka–Koga model to the WHO endpoint. **Results**: LR consistently outperformed XGBoost. The optimal Masaoka–Koga model combined Eph receptor A6 (EphA6) membranous, Yes-associated protein (YAP) nuclear, and histone deacetylase 4 (HDAC4) cytoplasmic H-scores, achieving an area under the curve (AUC) of 0.756. The optimal WHO model combined transcriptional coactivator with PDZ-binding motif (TAZ) cytoplasmic, EphA6 membranous, and YAP nuclear H-scores, achieving an AUC of 0.936. The Masaoka–Koga triad predicted WHO risk group with an AUC of 0.901. No tetrad improved trivariate performance. **Conclusions**: IHC H-score profiling combined with supervised ML identifies biologically interpretable candidate signatures for TET classification, although prospective external validation is required before clinical application.

## 1. Introduction

Thymic epithelial tumors (TETs) are rare, yet the most common primary tumors of the anterior mediastinum, and they present a formidable diagnostic and prognostic challenge [1]. Their biological behavior spans an unusually wide spectrum, from indolent, encapsulated thymomas that may be cured by surgery alone to aggressive thymic carcinomas with poor long-term outcomes [2,3]. Two parallel classification frameworks govern clinical decision-making. The Masaoka–Koga staging system assesses anatomical disease extent, distinguishing early stage (I–IIb), amenable to complete surgical resection, from advanced stage (III–IVb), which involves local invasion or distant spread and directly determines surgical approach and extent of resection [4]. The World Health Organization (WHO) histological classification groups TETs into subtypes A, AB, B1, B2, B3, and thymic carcinoma, each carrying independent prognostic weight [2]. Types B2, B3, and carcinoma form a high-risk group with markedly worse five-year survival than types A, AB, and B1, and this distinction governs the need for adjuvant chemotherapy and radiotherapy [5,6].

The difficulty is that both classification tasks are harder in practice than they appear in principle. Masaoka–Koga staging requires intraoperative and radiological correlation that is subject to institutional variability, while WHO subtype assignment depends on expert morphological assessment with reported interobserver kappa values of 0.45–0.68 and reclassification rates of up to 57% in non-specialized settings [7]. These limitations motivate the development of objective, quantitative, tissue-based tools. IHC-based H-score profiling provides semi-quantitative measurements of protein expression and cellular localization in tissue sections and therefore represents an accessible source of quantitative molecular data that can be derived from routinely processed FFPE material [8]. Previous studies in this cohort have characterized H-score expression of Hippo pathway components [9], histone deacetylases [10], ephrin receptors [11], and immune checkpoint molecules [12], documenting associations between specific H-score patterns and both Masaoka–Koga stage and WHO subtype.

ML approaches have been applied to TET data predominantly through CT-based radiomics [13,14,15]. However, no study has applied supervised ML to IHC H-score data for either Masaoka–Koga stage or WHO risk group prediction, and the combinatorial predictive value of multiple IHC markers has not been systematically evaluated for either endpoint. The present pilot study addresses both gaps simultaneously, applying LR [16] and XGBoost [17] across hierarchical biomarker combinations for each endpoint, with a cross-endpoint analysis to assess whether the optimal signature for one classification system carries predictive value for the other.

## 2. Materials and Methods

### 2.1. Study Population

The study population comprised 91 patients with histologically confirmed TETs whose archival FFPE material was retrieved from the Department of Pathology of Evangelismos General Hospital, Athens, Greece. All patients had been diagnosed between 2009 and 2019 and had complete medical records available. The cohort comprised 52 females (57%) and 39 males (43.0%), with a median age of 62 years (IQR 48–74 in a range of 27–88 years). Two separate analytical cohorts were derived from this source population.

Masaoka–Koga cohort (*n* = 81): Patients were excluded if H-score measurements were unavailable or Masaoka–Koga staging data were incomplete. Stage was encoded as binary, with 0 denoting early stage (I–IIb) and 1 denoting advanced stage (III–IVb). Demographic characteristics of the two stage groups are summarized in Table 1.

WHO cohort (*n* = 89): Of the 91 patients in the source cohort, two with Micronodular thymoma subtype were excluded due to ambiguous WHO classification [2], yielding a final analytical cohort of 89 patients. Patients were classified as low-risk (types A, AB, B1) or high-risk (types B2, B3, thymic carcinoma) [5,6]. Demographic characteristics and subtype distribution of the two risk groups are summarized in Table 2.

Institutional review board approval was obtained from the National and Kapodistrian University of Athens, Athens, Greece (protocol no. 140, 27 June 2019). Informed consent was waived due to the retrospective nature of the study.

### 2.2. Immunohistochemical Analysis and H-Score Quantification

IHC staining and H-score quantification have been described in full in previous publications for each biomarker panel [9,10,11,12]. Briefly, staining was performed on FFPE sections using standard protocols, and H-scores were calculated in neoplastic epithelial cells as H-score = Σ (intensity × percentage of cells) [8], yielding a continuous 0–300 scale per subcellular compartment. A total of 19 biomarkers were evaluated, spanning four biological domains (Table 3).

Missing H-score values were handled by complete-case analysis on a per-biomarker basis. For each analysis, only patients with available H-score measurements for all biomarkers under evaluation were included. No imputation was performed. This approach accounts for the variable effective sample sizes observed across biomarker combinations (range 51 to 89 patients), which are reported alongside all results. As a direct consequence of this approach, the effective sample size varies across biomarker combinations and is reported as N in each table. Patients excluded from one combination due to a missing value for a specific biomarker may be included in a different combination where all required measurements are available.

### 2.3. Machine Learning Modeling and Evaluation Framework

Binary classification was performed using LR and XGBoost, implemented in Python v3.12.7 using scikit-learn v1.6.1 [16] and XGBoost library v3.2.0 [17]. LR was chosen for its interpretability in small clinical datasets while XGBoost was run in parallel to test whether non-linear threshold effects or feature interactions existed. XGBoost models were configured with the following hyperparameters, namely n_estimators = 200, max_depth = 2, learning_rate = 0.05, subsample = 0.8, colsample_bytree = 0.8. These settings were selected a priori based on published recommendations for small clinical datasets. Systematic hyperparameter tuning via grid search was not performed, as the small effective sample sizes at each train/test split would render cross-validated tuning unreliable. For each biomarker combination, 100 independent stratified random train/test splits were performed (70% training/30% test).

This study was prepared with reference to the TRIPOD + AI reporting guidelines for multivariable prediction models developed using artificial intelligence and machine learning methods. A completed TRIPOD + AI checklist is provided as Supplementary Figure S1.

### 2.4. Handling of Class Imbalance

The Masaoka–Koga cohort exhibited substantial class imbalance (early: *n* = 59, 72.8%; advanced: *n* = 22, 27.2%; ratio ~ 2.7:1), addressed using SMOTE [18], implemented via imbalanced-learn (v0.13.0), and applied exclusively to the training set at each iteration. The test set at each split consisted entirely of real, unmodified data, ensuring no synthetic samples were present during evaluation and avoiding data leakage. The WHO cohort was near-perfectly balanced (50.6%/49.4%) and applying oversampling to an already balanced dataset would have introduced artificial variation without clinical justification [19], and was not applied. An exception was made in the cross-endpoint analysis, where the Masaoka–Koga optimal triad was applied to the WHO endpoint using the same SMOTE pipeline as the primary Masaoka–Koga analysis. This methodological choice was made deliberately to ensure comparability between the two directions of the cross-endpoint test, prioritizing consistency of pipeline over optimization for the WHO cohort specifically.

### 2.5. Analysis Structure

**Figure 1:**
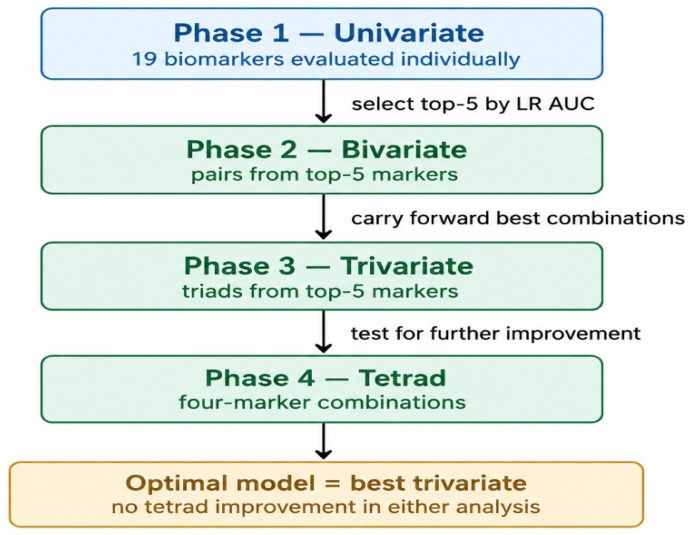
Hierarchical biomarker selection framework applied across both analyses. All 19 biomarkers were evaluated individually in Phase 1, and the top-5 performers by LR AUC were carried forward into bivariate, trivariate, and tetrad combinations, with each phase building on the results of the previous one. No tetrad combination improved upon the trivariate model in either analysis. SMOTE was applied in the Masaoka–Koga pipeline to address class imbalance. No oversampling was applied in the balanced WHO cohort.

Both analyses followed an identical four-phase hierarchical framework (Figure 1). The framework incorporated a nested feature selection procedure to eliminate potential optimism from global biomarker ranking. Specifically, in each of the 100 independent stratified train/test splits, the top-5 biomarkers were identified by univariate area under the curve (LR AUC) computed on the training partition only, with the held-out test set remaining completely unseen at this stage. The pre-specified multi-marker combinations evaluated in Phases 2, 3, and 4 correspond to those formed from the five biomarkers that emerged most consistently across all 100 nested rankings. Because these combinations are fixed independently of any individual test set outcome, no information from the test partition influenced the selection of combinations. Model performance was assessed using AUC with 95% confidence intervals derived from the distribution of AUC values across 100 splits, recall, which reflects the proportion of correctly classified advanced-stage cases, and specificity, which reflects the proportion of correctly classified early-stage cases. SMOTE [18] was applied in the Masaoka–Koga pipeline to address class imbalance, while no oversampling was applied in the balanced WHO cohort [19]. Decision Tree analyses (Supplementary Figures S2 and S3) are presented exclusively as exploratory visualizations of H-score thresholds for the optimal trivariate biomarker combinations. These trees were trained on the full analytical dataset without cross-validation and their training accuracy is optimistic. They carry no inferential weight and should not be interpreted as predictive models. All performance estimates reported in the main text are derived exclusively from the logistic regression framework with 100 independent stratified train/test splits.

Model calibration was assessed using the Brier score [20], computed as the mean squared error between predicted probabilities and observed binary outcomes, averaged across the 100 train/test splits. The Brier score ranges from 0 (perfect calibration) to 1, with a value of 0.25 representing the performance of a non-informative model on a balanced binary outcome. Median Brier scores are reported alongside AUC and recall for all multi-marker models.

Given the exploratory and hypothesis-generating nature of this study, formal correction for multiple comparisons was not applied. The hierarchical four-phase framework constrains the effective number of combinations evaluated at each phase, and no individual combination-level *p*-values are reported. All AUC estimates should be interpreted as descriptive performance metrics within the resampling framework rather than as results of formal hypothesis tests.

### 2.6. Statistical Software

All analyses were performed in Python 3.12.7. Machine learning models were implemented using scikit-learn (v1.6.1, last accessed on 17 May 2026) [16] and XGBoost library (v3.2.0, last accessed on 17 May 2026) [17]. SMOTE was applied using imbalanced-learn (v0.13.0, last accessed on 17 May 2026) [18]. Data manipulation was performed with pandas and NumPy (last accessed on 17 May 2026). The 95% confidence intervals for AUC were derived empirically as the 2.5th and 97.5th percentiles of the AUC distribution across 100 independent train/test splits using NumPy. Visualizations were generated with matplotlib and seaborn (last accessed on 17 May 2026). All code is available as a fully reproducible Jupyter notebook upon reasonable request to the corresponding author.

## 3. Results

### 3.1. Masaoka–Koga Stage Prediction

Descriptive statistics for the five top-performing biomarkers, stratified by Masaoka–Koga stage and by WHO risk group, are presented in Table 4 and Supplementary Figures S4–S7. EphA6 membranous, HDAC4 cytoplasmic (Masaoka–Koga stage), and TAZ cytoplasmic (WHO risk group) H-scores were higher in advanced-stage tumors, while YAP nuclear H-score was lower, consistent with previous findings from this cohort [9,10]. The directional pattern of EphA6 and YAP was preserved across both staging and histological risk group stratification, as shown in the right-hand columns of Table 4.

In the univariate analysis (Table 5), EphA6 membranous H-score achieved the highest LR AUC of 0.718, followed by HDAC4 cytoplasmic (0.672), TEAD4 cytoplasmic (0.656), and YAP nuclear (0.656). LR consistently outperformed XGBoost across all 19 markers, with the largest gap at the univariate level (ΔAUC = +0.111 for EphA6). The top-five markers advanced to bivariate analysis.

In the bivariate phase (Table 6), EphA6 membranous and YAP nuclear achieved the highest LR AUC of 0.750 (ΔAUC vs. univariate: +0.032), followed by EphA6 membranous and HDAC4 cytoplasmic (AUC 0.725). LR outperformed XGBoost in 9 of 10 pairs.

**Figure 2:**
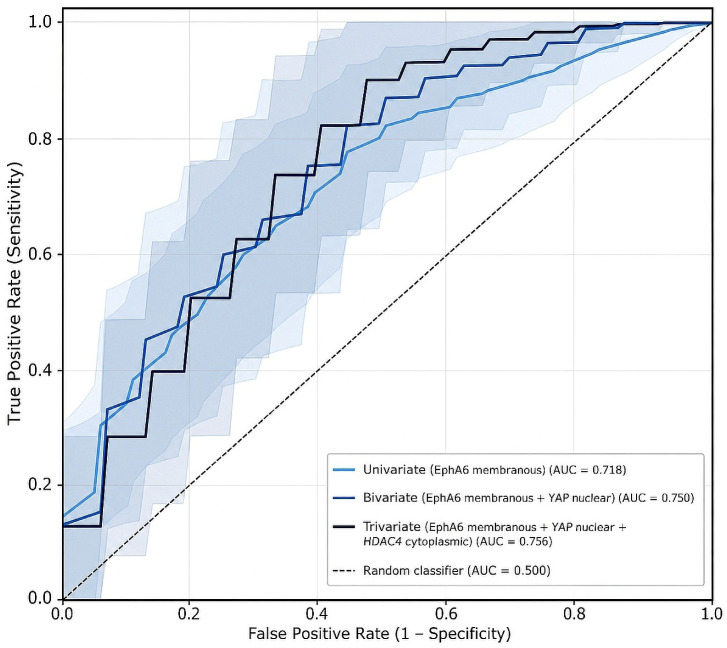
Mean ROC curves across 100 independent train/test splits (LR + SMOTE) for the best Masaoka–Koga model at each hierarchical level: univariate (EphA6 membranous, AUC 0.718), bivariate (EphA6 membranous + YAP nuclear, AUC 0.750), and trivariate (EphA6 membranous + YAP nuclear + HDAC4 cytoplasmic, AUC 0.756). Shaded bands represent ±1 standard deviation of the ROC curves across the 100 independent train/test splits, illustrating the variability of model performance.

In the trivariate phase (Table 7), EphA6 membranous, YAP nuclear, and HDAC4 cytoplasmic achieved the highest LR AUC of 0.756, which was the best model across all phases. LR outperformed XGBoost in all six triads. In the tetrad analysis (Table 8), no tetrad combination improved upon the trivariate model, as the best tetrad (EphA6 membranous and YAP nuclear and HDAC4 cytoplasmic and TAZ cytoplasmic) achieved AUC 0.738, representing a decrease of 0.018 relative to the trivariate model. ROC curves across all hierarchical levels are shown in Figure 2.

The improvement from the optimal bivariate model (AUC 0.750) to the optimal trivariate model (AUC 0.756) is modest in absolute terms. The primary contribution of HDAC4 cytoplasmic as a third predictor is an improvement in recall for the advanced-stage class rather than a substantial increase in overall discriminatory ability.

### 3.2. WHO Histological Risk Group Prediction

In the WHO analysis, LR outperformed XGBoost in 29 of 39 evaluated combinations (74.4%). Table 9 shows the top-eight univariate performers. TAZ cytoplasmic was the strongest individual predictor (AUC 0.840), followed by EphA6 membranous (0.828), YAP nuclear (0.803), and TEAD4 cytoplasmic (0.802). Three of the four top-ranked markers are Hippo pathway components. Descriptive H-score distributions by WHO risk group and by subtype are shown in Supplementary Figures S6 and S7.

YAP cytoplasmic H-scores were additionally compared between risk groups to assess evidence for nuclear-to-cytoplasmic redistribution. Median cytoplasmic YAP H-score was 0 in both low-risk (IQR 0–38) and high-risk (IQR 0–78) groups, with no systematic increase accompanying the reduction in nuclear YAP. Spearman correlation between nuclear and cytoplasmic YAP H-scores was positive (r = 0.363, *p* = 0.001), further arguing against a reciprocal redistribution pattern.

In the bivariate phase (Table 10), TAZ cytoplasmic and EphA6 membranous achieved the highest LR AUC of 0.903 (ΔAUC vs. univariate: +0.063). LR outperformed XGBoost in eight of 10 pairs.

**Figure 3:**
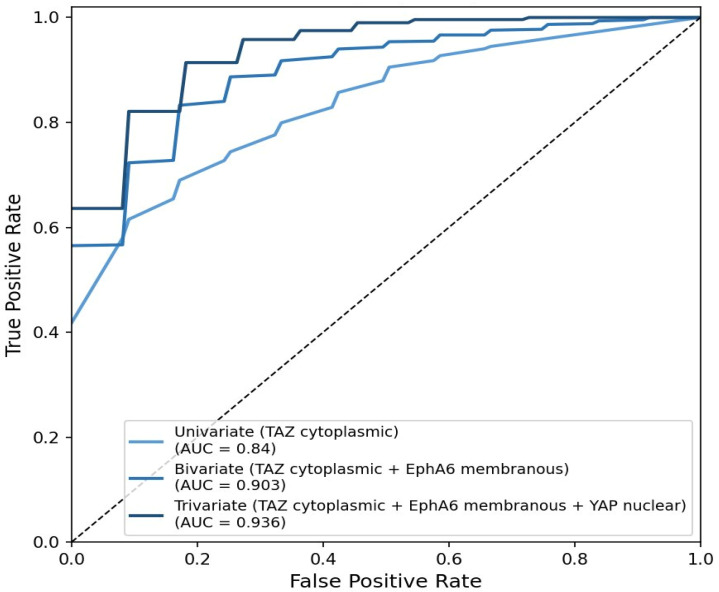
Mean ROC curves across 100 independent train/test splits (LR, no SMOTE) for the best WHO model at each hierarchical level: univariate (TAZ cytoplasmic, AUC 0.840), bivariate (TAZ + EphA6 membranous, AUC 0.903), and trivariate (TAZ cytoplasmic + EphA6 membranous + YAP nuclear, AUC 0.936).

In the trivariate phase (Table 11), TAZ cytoplasmic combined with EphA6 membranous and YAP nuclear was the best-performing model for both LR (AUC 0.936) and XGBoost (AUC 0.909). In the tetrad analysis (Table 12), no tetrad improved upon the trivariate model (best tetrad ΔAUC = −0.009). ROC curves across all hierarchical levels are shown in Figure 3.

### 3.3. Cross-Endpoint Analysis and LR Coefficient Comparison

The Masaoka–Koga optimal triad (EphA6 membranous + YAP nuclear + HDAC4 cytoplasmic) applied to the WHO endpoint in 72 patients achieved LR AUC 0.901 (recall 0.818), substantially higher than its primary Masaoka–Koga performance (AUC 0.756) despite identical analytical procedure on a partially overlapping sample. Table 13 summarizes all primary and cross-endpoint results. The directional gradient of the Masaoka–Koga triad markers across WHO histological subtypes is shown in Supplementary Figure S8. To explore the degree of biological convergence between the two classification systems, the optimal Masaoka–Koga trivariate model was applied to the WHO endpoint within the same largely overlapping cohort. This analysis is strictly exploratory and does not constitute independent or external validation, given the substantial patient overlap between the two analytical cohorts.

Table 14 presents the events per variable ratio (EPV) [21] for both optimal trivariate models, addressing the relationship between sample size and model complexity.

**Figure 4:**
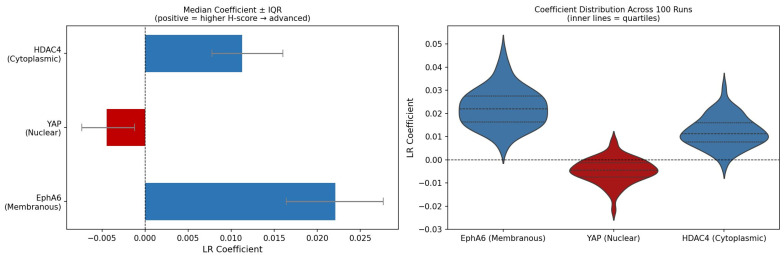
LR coefficients for the optimal Masaoka–Koga trivariate model (EphA6 membranous + YAP nuclear + HDAC4 cytoplasmic) across 100 iterations. **Left**: median ± IQR. **Right**: coefficient distribution (violin plot). All IQRs are wholly on one side of zero, confirming directional stability.

**Figure 5:**
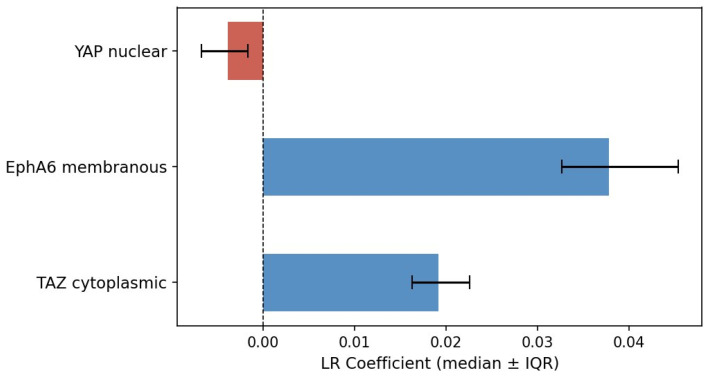
LR coefficients for the optimal WHO trivariate model (TAZ cytoplasmic + EphA6 membranous + YAP nuclear) across 100 splits. EphA6 membranous carries the largest positive coefficient; YAP nuclear is the only negative coefficient. All IQRs are wholly on one side of zero.

**Figure 6:**
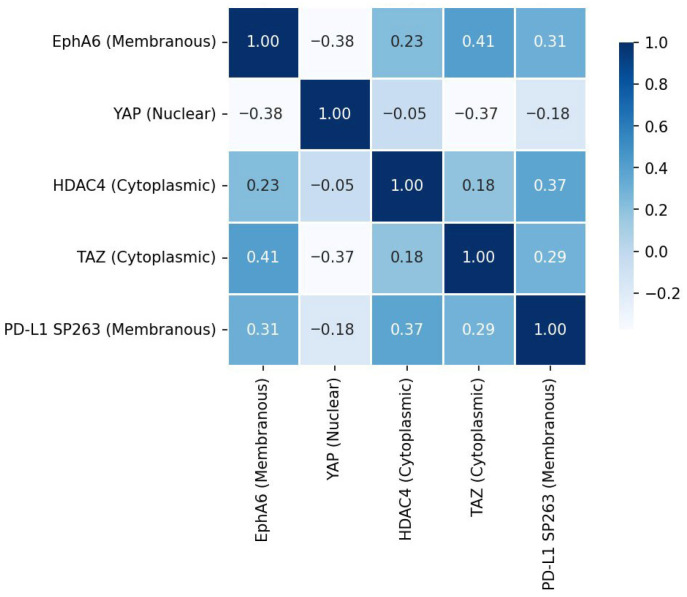
Spearman rank correlation matrix for the five top-performing Masaoka–Koga biomarkers. Maximum pairwise |r| = 0.40, confirming the absence of multicollinearity.

**Figure 7:**
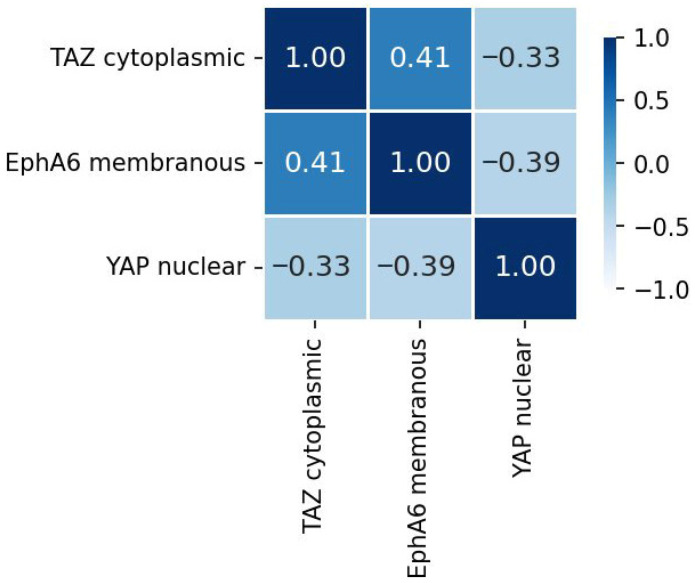
Spearman rank correlation matrix for the three optimal WHO trivariate markers. Maximum pairwise |r| = 0.41, confirming the absence of multicollinearity.

Table 15 presents the LR coefficient analysis for both optimal models. Two patterns are immediately apparent: EphA6 membranous and YAP nuclear appear in both models with directionally consistent coefficients (positive EphA6, negative YAP), and the absolute magnitude of both coefficients is larger in the WHO model, consistent with its higher overall discriminative performance. Spearman correlations confirmed the absence of multicollinearity in both models (maximum |r| = 0.40 for Masaoka–Koga, maximum |r| = 0.41 for WHO). Coefficient plots are illustrated in Figure 4 and Figure 5 and Spearman matrices in Figure 6 and Figure 7.

## 4. Discussion

### 4.1. Principal Finding

The main finding of this study is that IHC H-score profiling combined with supervised ML identifies biologically interpretable candidate signatures for both Masaoka–Koga stage and WHO histological risk group prediction in TETs. The two optimal models, EphA6 membranous combined with YAP nuclear and HDAC4 cytoplasmic for Masaoka–Koga staging (AUC 0.756), and TAZ cytoplasmic combined with EphA6 membranous and YAP nuclear for WHO risk group (AUC 0.936), share two common components with directionally consistent coefficients. The cross-endpoint result, namely the Masaoka–Koga triad achieving AUC 0.901 for WHO risk group prediction is consistent with the hypothesis that this shared biological signal operates across both classification systems, though this interpretation requires confirmation in independent cohorts. Two independent prognostic frameworks, one anatomical and one histological, converge on the same molecular markers as dominant predictors of TET aggressiveness. That convergence implies that it is unlikely to reflect a chance finding within this cohort alone, though external validation is required to exclude this possibility.

### 4.2. Shared Markers—EphA6 and YAP

EphA6 membranous H-score emerged as the strongest individual predictor of Masaoka–Koga stage (AUC 0.718) and the second-strongest for WHO risk group (AUC 0.828). Moreover, it revealed the largest LR coefficient in the WHO model (+0.0378). EphA6 belongs to the Eph receptor family, the largest family of receptor tyrosine kinases, which mediates contact-dependent bidirectional signaling involved in cell migration, invasion, and tumor progression [22,23,24]. Its elevated H-score in both advanced-stage and high-risk tumors, together with the known involvement of Eph receptor signaling in cancer cell communication, invasion, and progression, suggests EphA6 as a candidate consistently predictive biomarker across both endpoints in this cohort.

YAP nuclear H-score carried a negative LR coefficient in both models, with lower nuclear YAP associated with both advanced Masaoka–Koga stage and high-risk WHO classification. This finding is directly consistent with previous IHC data from this cohort [9], where nuclear YAP expression was elevated in the most biologically benign subtypes and lower in more aggressive tumors. The observed decrease in nuclear YAP H-score in high-risk tumors was not accompanied by a proportional increase in cytoplasmic YAP H-score. The median cytoplasmic YAP H-score was zero in both risk groups, arguing against a simple and stable nuclear-to-cytoplasmic redistribution mechanism. In other tumor types, reduced YAP expression has been associated with YAP1 promoter hypermethylation in breast cancer [25] and with FBXW7-mediated YAP ubiquitination and proteasomal degradation in hepatocellular carcinoma [26]. Moreover, Hippo pathway activation has been shown to increase LATS1 and YAP phosphorylation and restrict YAP nuclear localization in lung cancer cells [27], while coordinated LATS- and CK1-mediated phosphorylation can promote SCFβ-TRCP-dependent YAP ubiquitination and proteasomal degradation [28]. Thus, reduced nuclear YAP in aggressive TETs may reflect YAP1 downregulation or Hippo-associated proteasomal turnover, warranting validation of YAP1 mRNA, p-YAP, p-LATS1, and YAP stability. The inverse association was directionally stable across all 100 splits in both analyses without a single coefficient reversal, confirming a genuine biological signal.

A reciprocal observation supports this interpretation. The optimal WHO triad (TAZ cytoplasmic, EphA6 membranous, and YAP nuclear), when examined within the Masaoka–Koga analytical pipeline (Table 7), achieved LR AUC of 0.729, comparable to the Masaoka–Koga optimal triad performance of 0.756. The two pipelines differ in their use of SMOTE [18], which was justified by the imbalanced Masaoka–Koga cohort but was not applied to the balanced WHO cohort following [19]. Despite this methodological asymmetry, the comparable performance is consistent with a shared underlying molecular signal that both triads capture across the two classification systems.

### 4.3. Endpoint-Specific Markers—HDAC4 and TAZ

The divergence between the two models is equally informative. HDAC4 cytoplasmic appears in the Masaoka–Koga optimal triad (positive coefficient, +0.0113) but not in the WHO optimal model, where TAZ cytoplasmic takes its place as the strongest individual predictor (AUC 0.840, coefficient +0.0192). HDAC4 belongs to class IIa histone deacetylases, characterized by nuclear–cytoplasmic shuttling in response to cellular signaling, with cytoplasmic accumulation documented in advanced-stage TETs [10,29]. TAZ, the paralogue of YAP [30] and a central effector of the Hippo cascade [31,32], showed a dramatically different distributional pattern across WHO risk groups (median H-score 5.0 in low-risk vs. 200.0 in high-risk), a near-categorical biological distinction. The specificity of HDAC4 for anatomical staging and TAZ for histological risk group suggests that while EphA6 and YAP capture shared axes of TET aggressiveness, the two classification systems diverge in which additional molecular dimension is most discriminative, namely HDAC-associated regulation for anatomical staging and Hippo effector accumulation for histological risk.

### 4.4. LR vs. XGBoost—Consistent Pattern Across Both Analyses

LR held the advantage at every hierarchical level in both analyses. XGBoost is built for non-linearity [17] and excels when outcomes depend on threshold effects or feature interactions. The consistent superiority of LR across the majority of biomarker combinations is compatible with predominantly linear associations between H-score values and clinical outcome in this dataset. However, this finding should not be interpreted as evidence against the existence of non-linear interactions, which may require larger sample sizes or more exhaustive hyperparameter optimization to detect reliably [33,34]. The practical implication is the same in both cases, as LR coefficients map directly to log-odds ratios, making the model something a clinician can inspect, interrogate, and if necessary, dispute [35].

### 4.5. Comparison with Existing ML Studies in TETs

CT-based radiomics models for TET risk stratification have reported AUC values of 0.80–0.96 in multicenter cohorts [13,14,15]. The WHO analysis AUC of 0.936 is competitive with these figures, while the Masaoka–Koga AUC of 0.756 is lower, consistent with the smaller sample, class imbalance requiring SMOTE correction, and the greater biological complexity of anatomical staging. The key distinction is interpretability, as each of the markers across the two models is directly linked to a specific biological process independently characterized in the same tumor type [9,10,11]. ML is not uncovering patterns invisible to conventional analysis. It is reconfirming, with a more systematic methodology, what previous IHC studies in this cohort had already pointed to. Previous IHC H-score-based analysis in TETs has demonstrated prognostic value for individual markers [36], but systematic multi-marker ML evaluation has not been attempted. No publicly available TET dataset with matched IHC H-score measurements and staging or subtype data exists, as genomic repositories provide transcript-level data not interchangeable with protein-level H-scores, given that fewer than 20–25% of genes show statistically significant mRNA–protein concordance in a variety of tumors [37,38].

### 4.6. Limitations

The principal limitation of both analyses is sample size, as the Masaoka–Koga model was built on 81 patients (22 advanced-stage events) and the WHO model on 67 complete cases for the optimal triad (approximately 33 per class), both leaving a narrow margin against the widely cited 10 events per variable heuristic (EPV) [21]. The optimal Masaoka–Koga trivariate model (three predictors, 22 advanced-stage events) yields EPV = 7.3, below the commonly cited threshold of 10, indicating that coefficient estimates may be unstable. This is partially mitigated by the use of 100 independent stratified splits and by the observed directional stability of all logistic regression coefficients across all 100 splits. The WHO analysis (EPV = 11.0) meets the conventional threshold. Both models should be interpreted as hypothesis-generating and require external validation before clinical application. Because biomarker selection and final model evaluation were performed within the same retrospective cohort, the reported AUC values should be interpreted as internally optimized estimates rather than externally validated performance metrics. The internal stability achieved across 100 train/test splits in each analysis, with no coefficient reversals in either, provides supportive evidence for model robustness, although it does not replace external validation [20]. Calibration using the Hosmer–Lemeshow test has limited power in small samples [39] and any non-significant result would have been uninformative. Decision Tree analyses were fitted on full datasets without cross-validation. The present machine learning analysis should be understood as a methodological extension of the existing biomarker characterization work rather than as an independent biological replication.

### 4.7. Future Directions

The absence of external validation on an independent cohort is the primary limitation of this study. All reported AUC estimates reflect internal performance within the single-center Evangelismos Hospital series and cannot be directly extrapolated to other populations. The rarity of thymic epithelial tumors renders single-institution external validation practically unfeasible within a reasonable timeframe. Multicenter validation, ideally through established international networks, is required before any clinical application of the proposed models. The present study should therefore be interpreted strictly as hypothesis-generating [20,40]. A larger dataset would permit survival analysis linking both signatures to time-to-recurrence outcomes and calibration assessment [41], testing whether the EphA6/YAP thresholds from the Decision Tree analyses reproduced across institutions and investigating whether HDAC4 and TAZ provide complementary or interchangeable information across the two endpoints. The convergence of EphA6 and YAP across both analyses provides a biologically motivated starting point for a prospective targeted IHC panel.

## 5. Conclusions

IHC H-score profiling combined with supervised ML identifies candidate signatures for both Masaoka–Koga stage and WHO histological risk group prediction in TETs. The Masaoka–Koga optimal model (EphA6 membranous, YAP nuclear, and HDAC4 cytoplasmic) achieved AUC 0.756, and the WHO optimal model (TAZ cytoplasmic, EphA6 membranous, and YAP nuclear) achieved AUC 0.936, across 100 independent train/test splits in each analysis. LR outperformed XGBoost in both analyses, supporting predominantly additive biomarker–outcome relationships. EphA6 membranous and YAP nuclear appeared in both optimal models with directionally consistent, coefficient-stable associations, confirmed without a single directional reversal across all splits, and the Masaoka–Koga triad predicted WHO risk group with AUC 0.901 in the cross-endpoint analysis. The three markers in each model capture independent biological signaling axes. No tetrad combination improved upon the trivariate performance in either analysis. External validation in a prospective, multi-institutional cohort is the necessary next step.

## Figures and Tables

**Table 1:** Demographic characteristics of the Masaoka–Koga analytical cohort (*n* = 81), stratified by stage group. Interquartile Range (IQR) is the statistical measure that describes the spread or variability of the data around the median.

Characteristic	Early Stage (I–IIb)	Advanced Stage (III–IVb)
Patients, *n* (%)	59 (72.8%)	22 (27.2%)
Median age, years (IQR)	60 (46–71)	66 (50–74)
Female, *n* (%)	35 (59.3%)	9 (40.9%)
Male, *n* (%)	24 (40.7%)	13 (59.1%)
Stage distribution, *n* (%)	I: 13 (16.0%); IIa: 32 (39.5%); IIb: 14 (17.3%)	III: 16 (19.8%); IVa: 3 (3.7%); IVb: 3 (3.7%)

**Table 2:** Demographic characteristics and WHO subtype distribution of the WHO analytical cohort (*n* = 89), stratified by histological risk group. Interquartile Range (IQR) is the statistical measure that describes the spread or variability of the data around the median.

Characteristic	Low-Risk (A/AB/B1)	High-Risk (B2/B3/Carcinoma)
Patients, *n* (%)	45 (50.6%)	44 (49.4%)
Median age, years (IQR)	65 (52–73)	58 (44–74)
Female, *n* (%)	27 (60.0%)	22 (50.0%)
Male, *n* (%)	18 (40.0%)	22 (50.0%)
Subtype distribution, *n* (%)	A: 12 (26.7%); AB: 19 (42.2%); B1: 14 (31.1%)	B2: 19 (43.2%); B3: 14 (31.8%); carcinoma: 11 (25.0%)

**Table 3:** The 19 IHC H-score biomarkers evaluated across both analyses, grouped by signaling pathway and subcellular compartment. Asterisks (*) indicate markers that appear as components of either optimal trivariate model.

Hippo Pathway	Histone Deacetylases	Ephrin Receptors	Immune Checkpoints
TEAD4 (cytoplasmic)	HDAC1 (nuclear)	EphA2 (membranous)	PD-L1 SP142 (membranous)
TEAD4 (nuclear)	HDAC2 (cytoplasmic)	EphA6 (membranous) *	PD-L1 SP263 (membranous)
LATS1 (cytoplasmic)	HDAC3 (cytoplasmic)		
LATS1 (nuclear)	HDAC3 (nuclear)		
YAP (cytoplasmic)	HDAC4 (cytoplasmic) *		
YAP (nuclear) *	HDAC5 (cytoplasmic)		
TAZ (cytoplasmic) *	HDAC6 (cytoplasmic)		
TAZ (nuclear)			

**Table 4:** Descriptive statistics of the five top-performing biomarkers stratified by Masaoka–Koga stage. Right-hand columns show the corresponding WHO risk group medians for EphA6 membranous, YAP nuclear, and TAZ cytoplasmic, illustrating the concordance of directional associations across both endpoints. (—) indicates markers not selected for the WHO optimal model. The marker *n* represents the number of cases expressing the biomarker according to each stage. Interquartile Range (IQR) is the statistical measure that describes the spread or variability of the data around the median.

Biomarker (Compartment)	Masaoka–Koga Stage		Masaoka–Koga H-Score		WHO Risk Group H-Score	
	**Stage Group**	***n***	**Median**	**IQR**	**Median**	**IQR**
EphA6 (membranous)	Early (I–IIb)	59	30	5–80	25 (low-risk)	5.0–67.5
	Advanced (III–IVb)	22	80	52.5–140	80 (high-risk)	70.0–140.0
YAP (nuclear)	Early (I–IIb)	52	30	10–140	80 (low-risk)	21.2–147.5
	Advanced (III–IVb)	18	10	0–48.8	10 (high-risk)	0.0–20.0
TAZ (cytoplasmic)	Early (I–IIb)	52	65	0–125	5 (low-risk)	0.0–90.0
	Advanced (III–IVb)	19	100	80–200	200 (high-risk)	100.0–200.0
HDAC4 (cytoplasmic)	Early (I–IIb)	56	20	0–60	—	—
	Advanced (III–IVb)	22	65	17.5–97.5	—	—
PD-L1 SP263	Early (I–IIb)	59	100	0–109.5	—	—
	Advanced (III–IVb)	22	100	100–145	—	—

**Table 5:** Masaoka–Koga stage prediction—univariate analysis (LR + SMOTE vs. XGBoost + SMOTE, 100 stratified splits). All 19 biomarkers ranked by LR AUC. The 95% CI derived from the 2.5th–97.5th percentiles of the AUC distribution. Brier score: mean squared error of probabilistic predictions; values below 0.25 indicate improvement over a non-informative model. ΔAUC = LR AUC minus XGBoost AUC. Specificity is also reported for the LR model.

Biomarker (Compartment)	LR + SMOTE					XGBoost + SMOTE		Performance Gap	
	AUC	95% CI	Recall	Brier	Specificity	AUC	Recall	ΔAUC	N
EphA6 (membranous)	0.718	[0.546–0.881]	0.714	0.210	0.611	0.607	0.571	+0.111	59/22
HDAC4 (cytoplasmic)	0.672	[0.413–0.853]	0.714	0.237	0.706	0.613	0.429	+0.059	56/22
TEAD4 (cytoplasmic)	0.656	[0.422–0.870]	0.750	0.242	0.583	0.578	0.250	+0.078	—
YAP (nuclear)	0.656	[0.427–0.895]	0.700	0.242	0.469	0.606	0.600	+0.050	52/18
TAZ (cytoplasmic)	0.651	[0.440–0.846]	0.500	0.237	0.750	0.589	0.500	+0.062	52/19
HDAC5 (cytoplasmic)	0.641	[0.396–0.856]	0.400	0.234	0.706	0.562	0.400	+0.079	—
PD-L1 SP263 (membranous)	0.617	[0.390–0.782]	0.857	0.245	0.389	0.550	0.571	+0.067	59/22
HDAC6 (cytoplasmic)	0.577	[0.220–0.784]	0.500	0.248	0.714	0.543	0.500	+0.034	—
HDAC1 (nuclear)	0.576	[0.202–0.744]	0.571	0.249	0.353	0.597	0.571	−0.021	—
HDAC2 (cytoplasmic)	0.562	[0.193–0.783]	0.571	0.246	0.714	0.540	0.571	+0.022	—
HDAC3 (cytoplasmic)	0.554	[0.200–0.704]	0.571	0.250	0.923	0.530	0.429	+0.024	—
EphA2 (membranous)	0.544	[0.208–0.715]	0.500	0.250	0.529	0.528	0.500	+0.016	—
LATS1 (nuclear)	0.538	[0.164–0.685]	0.500	0.252	0.750	0.522	0.500	+0.016	—
TEAD4 (nuclear)	0.530	[0.182–0.709]	0.500	0.252	0.500	0.508	0.500	+0.022	—
TAZ (nuclear)	0.527	[0.265–0.639]	0.500	0.248	0.688	0.504	0.500	+0.023	—
YAP (cytoplasmic)	0.522	[0.218–0.657]	0.500	0.253	0.538	0.504	0.500	+0.018	—
PD-L1 SP142 (membranous)	0.519	[0.278–0.655]	0.571	0.253	0.444	0.500	0.500	+0.019	—
HDAC3 (nuclear)	0.513	[0.219–0.589]	0.500	0.254	0.467	0.496	0.500	+0.017	—
LATS1 (cytoplasmic)	0.501	[0.200–0.704]	0.500	0.256	0.467	0.492	0.500	+0.009	—

**Table 6:** Masaoka–Koga stage prediction—bivariate analysis (LR + SMOTE vs. XGBoost + SMOTE, 100 stratified splits). All C(5,2) = 10 pre-specified pairs from the top-five biomarkers, ranked by LR AUC.

Biomarker (Compartment)	LR + SMOTE					XGBoost + SMOTE		Performance Gap	
	AUC	95% CI	Recall	Brier	Specificity	AUC	Recall	ΔAUC	N
EphA6 (membranous) + YAP (nuclear)	0.750	[0.518–0.907]	0.600	0.196	0.688	0.684	0.600	+0.066	52/18
EphA6 (membranous) + HDAC4 (cytoplasmic)	0.725	[0.538–0.914]	0.571	0.219	0.676	0.659	0.571	+0.066	56/22
EphA6 (membranous) + PD-L1 SP263 (membranous)	0.704	[0.515–0.859]	0.643	0.213	0.667	0.637	0.571	+0.067	59/22
EphA6 (membranous) + TAZ (cytoplasmic)	0.693	[0.465–0.870]	0.667	0.218	0.688	0.648	0.500	+0.045	52/19
HDAC4 (cytoplasmic) + TAZ (cytoplasmic)	0.667	[0.447–0.828]	0.500	0.242	0.667	0.612	0.500	+0.055	52/19
YAP (nuclear) + TAZ (cytoplasmic)	0.643	[0.405–0.847]	0.600	0.238	0.643	0.609	0.500	+0.034	52/18
HDAC4 (cytoplasmic) + YAP (nuclear)	0.636	[0.435–0.845]	0.500	0.245	0.600	0.590	0.500	+0.046	52/18
HDAC4 (cytoplasmic) + PD-L1 SP263 (membranous)	0.634	[0.361–0.817]	0.571	0.244	0.647	0.586	0.500	+0.048	56/22
TAZ (cytoplasmic) + PD-L1 SP263 (membranous)	0.628	[0.404–0.792]	0.500	0.242	0.625	0.580	0.500	+0.048	52/19
YAP (nuclear) + PD-L1 SP263 (membranous)	0.609	[0.324–0.835]	0.600	0.242	0.500	0.561	0.500	+0.048	52/18

**Table 7:** Masaoka–Koga stage prediction—trivariate analysis (LR + SMOTE vs. XGBoost + SMOTE, 100 stratified splits). Six pre-specified triads anchored on EphA6 (membranous), ranked by LR AUC. Reference model: EphA6 + YAP (nuclear) + HDAC4 (cytoplasmic), LR AUC = 0.756 (95% CI [0.550–0.912]), Brier = 0.206, Specificity = 0.733.

Biomarker (Compartment)	LR + SMOTE					XGBoost + SMOTE		Performance Gap	
	AUC	95% CI	Recall	Brier	Specificity	AUC	Recall	ΔAUC	N
EphA6 (membranous) + YAP (nuclear) + HDAC4 (cytoplasmic)	0.756	[0.550–0.912]	0.667	0.206	0.733	0.694	0.571	+0.062	56/22
EphA6 (membranous) + YAP (nuclear) + PD-L1 SP263 (membranous)	0.738	[0.484–0.897]	0.600	0.206	0.688	0.671	0.571	+0.067	59/22
EphA6 (membranous) + YAP (nuclear) + TAZ (cytoplasmic)	0.729	[0.471–0.893]	0.600	0.212	0.714	0.681	0.600	+0.048	52/19
EphA6 (membranous) + HDAC4 (cytoplasmic) + PD-L1 SP263	0.702	[0.538–0.883]	0.571	0.227	0.706	0.655	0.571	+0.047	56/22
EphA6 (membranous) + HDAC4 (cytoplasmic) + TAZ (cytoplasmic)	0.678	[0.461–0.856]	0.667	0.231	0.667	0.631	0.500	+0.047	52/19
EphA6 (membranous) + TAZ (cytoplasmic) + PD-L1 SP263	0.646	[0.421–0.839]	0.500	0.230	0.688	0.619	0.500	+0.027	52/19

**Table 8:** Masaoka–Koga stage prediction—tetrad verification (LR + SMOTE, 100 stratified splits). All C(5,4) = 5 tetrads from the top-five pool. ΔAUC = tetrad AUC minus reference triad AUC (0.756). No tetrad improved upon the trivariate model.

Biomarker (Compartment)	LR AUC	95% CI	ΔAUC	Recall	Brier	Specificity	N
EphA6 (membranous) + YAP (nuclear) + HDAC4 (cytoplasmic) + TAZ (cytoplasmic)	0.738	[0.491–0.913]	−0.018	0.600	0.216	0.733	52/19
EphA6 (membranous) + YAP (nuclear) + HDAC4 (cytoplasmic) + PD-L1 SP263	0.722	[0.522–0.895]	−0.034	0.500	0.217	0.692	56/22
EphA6 (membranous) + YAP (nuclear) + PD-L1 SP263 + TAZ (cytoplasmic)	0.721	[0.477–0.904]	−0.035	0.600	0.220	0.714	52/19
EphA6 (membranous) + HDAC4 (cytoplasmic) + PD-L1 SP263 + TAZ (cytoplasmic)	0.656	[0.466–0.833]	−0.100	0.500	0.242	0.667	52/19
YAP (nuclear) + HDAC4 (cytoplasmic) + PD-L1 SP263 + TAZ (cytoplasmic)	0.620	[0.384–0.839]	−0.136	0.500	0.256	0.615	52/18

**Table 9:** WHO histological risk group prediction—univariate analysis (LR vs. XGBoost, 100 stratified splits, no SMOTE). All 19 biomarkers ranked by LR AUC. The 95% CI derived from the 2.5th–97.5th percentiles of the AUC distribution. Brier score: lower values indicate better calibration; null model Brier = 0.25 for a balanced binary outcome. ΔAUC = LR AUC minus XGBoost AUC. Specificity is also reported for the LR model.

Biomarker (Compartment)	LR					XGBoost		Performance Gap	
	AUC	95% CI	Recall	Brier	Specificity	AUC	Recall	ΔAUC	N
TAZ (cytoplasmic)	0.840	[0.724–0.941]	0.750	0.164	0.750	0.790	0.750	+0.050	77
EphA6 (membranous)	0.828	[0.692–0.955]	0.769	0.162	0.786	0.821	0.692	+0.007	89
YAP (nuclear)	0.803	[0.599–0.927]	0.833	0.203	0.583	0.763	0.750	+0.040	76
TEAD4 (cytoplasmic)	0.802	[0.594–0.931]	0.778	0.190	0.625	0.715	0.667	+0.087	56
PD-L1 SP142 (membranous)	0.717	[0.596–0.868]	0.769	0.219	0.714	0.731	0.615	−0.014	89
HDAC5 (cytoplasmic)	0.700	[0.541–0.877]	0.583	0.220	0.792	0.602	0.500	+0.098	80
PD-L1 SP263 (membranous)	0.692	[0.530–0.837]	0.846	0.220	0.500	0.670	0.769	+0.022	89
EphA2 (membranous)	0.665	[0.516–0.794]	0.615	0.233	0.571	0.573	0.500	+0.092	88
HDAC4 (cytoplasmic)	0.652	[0.426–0.835]	0.615	0.241	0.615	0.611	0.538	+0.041	84
LATS1 (nuclear)	0.617	[0.357–0.811]	0.600	0.242	0.556	0.494	0.550	+0.123	61
LATS1 (cytoplasmic)	0.600	[0.500–0.700]	0.200	0.223	1.000	0.500	1.000	+0.100	61
HDAC6 (cytoplasmic)	0.584	[0.416–0.727]	0.364	0.242	0.800	0.530	0.273	+0.054	69
HDAC2 (cytoplasmic)	0.564	[0.245–0.711]	0.636	0.245	0.400	0.595	0.636	−0.031	69
HDAC1 (nuclear)	0.559	[0.279–0.709]	0.692	0.249	0.308	0.565	0.385	−0.006	85
HDAC3 (nuclear)	0.545	[0.249–0.684]	0.583	0.248	0.500	0.552	0.583	−0.007	77
TAZ (nuclear)	0.519	[0.193–0.689]	0.292	0.254	0.667	0.590	0.500	−0.071	77
YAP (cytoplasmic)	0.489	[0.289–0.621]	0.250	0.253	0.652	0.593	0.250	−0.104	76
HDAC3 (cytoplasmic)	0.451	[0.278–0.590]	0.583	0.252	0.333	0.635	0.667	−0.184	77
TEAD4 (nuclear)	0.424	[0.292–0.507]	0.667	0.254	0.250	0.309	0.444	+0.115	56

**Table 10:** WHO histological risk group prediction—bivariate analysis (LR vs. XGBoost, 100 stratified splits). All C(5,2) = 10 pre-specified pairs from the top-five biomarkers, ranked by LR AUC.

Biomarker (Compartment)	LR					XGBoost		Performance Gap	
	AUC	95% CI	Recall	Brier	Specificity	AUC	Recall	ΔAUC	N
TAZ (cytoplasmic) + EphA6 (membranous)	0.903	[0.796–0.972]	0.833	0.131	0.833	0.858	0.750	+0.045	77
TAZ (cytoplasmic) + YAP (nuclear)	0.895	[0.740–0.982]	0.800	0.139	0.818	0.855	0.800	+0.040	67
EphA6 (membranous) + YAP (nuclear)	0.884	[0.771–0.977]	0.818	0.144	0.818	0.871	0.750	+0.013	76
EphA6 (membranous) + TEAD4 (cytoplasmic)	0.847	[0.645–0.958]	0.667	0.176	0.750	0.799	0.667	+0.048	56
TAZ (cytoplasmic) + PD-L1 SP142 (membranous)	0.840	[0.691–0.934]	0.750	0.163	0.833	0.816	0.750	+0.024	77
YAP (nuclear) + TEAD4 (cytoplasmic)	0.832	[0.628–0.977]	0.750	0.175	0.625	0.855	0.750	−0.023	51
TAZ (cytoplasmic) + TEAD4 (cytoplasmic)	0.828	[0.656–0.993]	0.750	0.169	0.750	0.773	0.750	+0.055	51
EphA6 (membranous) + PD-L1 SP142 (membranous)	0.828	[0.691–0.936]	0.769	0.166	0.786	0.824	0.692	+0.004	89
YAP (nuclear) + PD-L1 SP142 (membranous)	0.801	[0.655–0.944]	0.727	0.188	0.727	0.831	0.727	−0.030	76
TEAD4 (cytoplasmic) + PD-L1 SP142 (membranous)	0.774	[0.552–0.920]	0.667	0.202	0.688	0.740	0.778	+0.034	56

**Table 11:** WHO histological risk group prediction—trivariate analysis (LR vs. XGBoost, 100 stratified splits). All C(5,3) = 10 pre-specified triads, ranked by LR AUC. Reference model (bold row 1): TAZ (cytoplasmic) + EphA6 (membranous) + YAP (nuclear), LR AUC = 0.936 (95% CI [0.859–1.000]), Brier = 0.114, Specificity = 0.909.

Biomarker (Compartment)	LR					XGBoost		Performance Gap	
	AUC	95% CI	Recall	Brier	Specificity	AUC	Recall	ΔAUC	N
TAZ (cytoplasmic) + EphA6 (membranous) + YAP (nuclear)	0.936	[0.859–1.000]	0.800	0.114	0.909	0.909	0.800	+0.027	67
TAZ (cytoplasmic) + YAP (nuclear) + PD-L1 SP142 (membranous)	0.905	[0.731–0.996]	0.800	0.136	0.909	0.882	0.700	+0.023	67
TAZ (cytoplasmic) + EphA6 (membranous) + PD-L1 SP142 (membranous)	0.892	[0.785–0.966]	0.833	0.136	0.833	0.852	0.750	+0.040	77
EphA6 (membranous) + YAP (nuclear) + PD-L1 SP142 (membranous)	0.879	[0.750–0.981]	0.750	0.151	0.784	0.871	0.818	+0.008	76
TAZ (cytoplasmic) + YAP (nuclear) + TEAD4 (cytoplasmic)	0.878	[0.724–1.000]	0.857	0.144	0.714	0.913	0.857	−0.035	46
EphA6 (membranous) + YAP (nuclear) + TEAD4 (cytoplasmic)	0.875	[0.711–0.969]	0.750	0.166	0.750	0.898	0.875	−0.023	51
TAZ (cytoplasmic) + EphA6 (membranous) + TEAD4 (cytoplasmic)	0.844	[0.695–0.985]	0.812	0.163	0.875	0.820	0.750	+0.024	51
TAZ (cytoplasmic) + TEAD4 (cytoplasmic) + PD-L1 SP142 (membranous)	0.812	[0.641–0.977]	0.750	0.187	0.750	0.770	0.750	+0.042	51
EphA6 (membranous) + TEAD4 (cytoplasmic) + PD-L1 SP142 (membranous)	0.812	[0.604–0.958]	0.667	0.188	0.750	0.764	0.667	+0.048	56
YAP (nuclear) + TEAD4 (cytoplasmic) + PD-L1 SP142 (membranous)	0.797	[0.554–0.977]	0.750	0.187	0.625	0.875	0.875	−0.078	51

**Table 12:** WHO histological risk group prediction—tetrad verification (LR, 100 stratified splits). All C(5,4) = 5 tetrads from the top-five pool. ΔAUC = tetrad AUC minus reference triad AUC (0.936). No tetrad improved upon the trivariate model.

Biomarker (Compartment)	LR AUC	95% CI	ΔAUC	Recall	Brier	Specificity	N
TAZ (cytoplasmic) + EphA6 (membranous) + YAP (nuclear) + PD-L1 SP142 (membranous)	0.927	[0.813–0.996]	−0.009	0.800	0.125	0.818	67
TAZ (cytoplasmic) + EphA6 (membranous) + YAP (nuclear) + TEAD4 (cytoplasmic)	0.918	[0.734–1.000]	−0.018	0.857	0.125	0.857	46
TAZ (cytoplasmic) + YAP (nuclear) + TEAD4 (cytoplasmic) + PD-L1 SP142 (membranous)	0.857	[0.622–0.990]	−0.079	0.714	0.175	0.714	46
TAZ (cytoplasmic) + EphA6 (membranous) + TEAD4 (cytoplasmic) + PD-L1 SP142 (membranous)	0.844	[0.672–0.969]	−0.092	0.875	0.169	0.750	51
EphA6 (membranous) + YAP (nuclear) + TEAD4 (cytoplasmic) + PD-L1 SP142 (membranous)	0.844	[0.562–0.969]	−0.092	0.750	0.177	0.750	51

**Table 13:** Summary of primary and cross-endpoint results. Analytical cohort refers to the total cohort available for each classification endpoint, whereas complete cases used in model refers to patients with available H-score data for all biomarkers included in the corresponding trivariate model. SMOTE applied to training set only where indicated.

Model	Classification Endpoint	Analytical Cohort	Complete Cases Used in Model	LR AUC	SMOTE
EphA6 (membranous) + YAP (nuclear) + HDAC4 (cytoplasmic)	Masaoka–Koga (primary)	81	78 (56 early/22 advanced)	0.756	Yes
EphA6 (membranous) + YAP (nuclear) + HDAC4 (cytoplasmic)	WHO risk group (cross-endpoint)	89	72 (35 low-risk/37 high-risk)	0.901	No
TAZ (cytoplasmic) + EphA6 (membranous) + YAP (nuclear)	WHO risk group (primary)	89	67 (34 low-risk/33 high-risk)	0.936	No

**Table 14:** Events per variable (EPV) for both optimal trivariate models. Complete-case N reflects patients with non-missing H-score values for all three biomarkers in each model. EPV is calculated as the number of minority class events divided by the number of predictors (3).

Model	Complete-Case N	Minority Class Events	Majority Class Events	EPV
Masaoka–Koga trivariate (EphA6 membranous + YAP nuclear + HDAC4 cytoplasmic)	56	22 (advanced)	34 (early)	7.3
WHO trivariate (TAZ cytoplasmic + EphA6 membranous + YAP nuclear)	67	33 (high-risk)	34 (low-risk)	11.0

**Table 15:** LR coefficient analysis for both optimal trivariate models (median ± IQR, 100 splits). Rows 1–3: Masaoka–Koga model; rows 4–6: WHO model (shaded). EphA6 membranous and YAP nuclear appear in both models with consistent directional signs.

Biomarker	Endpoint	Median Coef.	Q25	Q75	Direction	Stable
EphA6 (membranous)	Masaoka (primary)	+0.0222	+0.0165	+0.0278	Positive	Yes
YAP (nuclear)	Masaoka (primary)	−0.0044	−0.0073	−0.0012	Negative	Yes
HDAC4 (cytoplasmic)	Masaoka (primary)	+0.0113	+0.0077	+0.0160	Positive	Yes
TAZ (cytoplasmic)	WHO (primary)	+0.0192	+0.0163	+0.0226	Positive	Yes
EphA6 (membranous)	WHO (primary)	+0.0378	+0.0327	+0.0454	Positive	Yes
YAP (nuclear)	WHO (primary)	−0.0039	−0.0068	−0.0017	Negative	Yes

## Data Availability

The data presented in this study are available on reasonable request from the corresponding author. The data are not publicly available due to privacy restrictions.

## Supplementary Materials

The following supporting information can be downloaded at https://www.mdpi.com/article/10.3390/diagnostics16132118/s1. Supplementary Figures S1–S8 are available as separate files accompanying this submission. S1: Completed TRIPOD+AI checklist for the present study, reporting compliance with the TRIPOD+AI guidelines for studies developing or evaluating multivariable prediction models using artificial intelligence and machine learning methods. S2: Exploratory Decision Tree for the optimal Masaoka-Koga trivariate model. S3: Exploratory Decision Tree for the optimal WHO trivariate model. S4: H-score distributions for the five top-performing Masaoka-Koga biomarkers stratified by Masaoka-Koga stage group (early I–IIb vs advanced III–IVb). S5: H-score distributions for the optimal Masaoka-Koga trivariate markers stratified across all six individual Masaoka-Koga stages. S6: H-score distributions for the optimal WHO trivariate markers stratified by WHO histological risk group. S7: H-score distributions for the optimal WHO trivariate markers stratified by WHO histological subtype. S8: H-score distributions for the optimal Masaoka-Koga trivariate markers stratified by WHO histological subtype, illustrating the cross-endpoint biological gradient.
