# Supplementary material for: Machine Learning-Based Prediction of Masaoka–Koga Stage and WHO Histological Risk Group in Thymic Epithelial Tumors Using Biomarker Combinations
Source: Diagnostics (Basel). 2026 Jul 7;16(13):2118. doi: 10.3390/diagnostics16132118 (PMC13360224; doi:10.3390/diagnostics16132118)
Supplement: Supplementary file 1 [file diagnostics-16-02118-s001.zip › Supplementary Figure S1.pdf]

TRIPOD+AI Checklist

Machine Learning-Based Prediction of Masaoka-Koga Stage and WHO Risk Group in Thymic Epithelial Tumors Using Immunohistochemical H-Score Profiles

D = development; E = evaluation; D;E = applicable to both. Page references correspond to the revised manuscript submitted in response to peer review.

Section/Topic	Item	D/E	Checklist item	Reported on page
**TITLE**				
**Title**	1	D;E	Identify the study as developing or evaluating the performance of a multivariable prediction model, the target population, and the outcome to be predicted	Title page (p.1)
**ABSTRACT**				
**Abstract**	2	D;E	See TRIPOD+AI for Abstracts checklist	Abstract (p.1)
**INTRODUCTION**				
**Background**	3a	D;E	Explain the healthcare context and rationale for developing the prediction model, including references to existing models	Introduction, paragraphs 1-2
	3b	D;E	Describe the target population and intended purpose of the prediction model in the context of the care pathway, including intended users	Introduction, final paragraph
	3c	D;E	Describe any known health inequalities between sociodemographic groups	Not assessed; single-center retrospective cohort without sociodemographic stratification (Limitations)
**Objectives**	4	D;E	Specify the study objectives, including whether the study describes development or validation of a prediction model (or both)	Introduction, final paragraph (model development only; no external validation)
**METHODS**				
**Data**	5a	D;E	Describe the sources of data separately for development and evaluation datasets, rationale, and representativeness	Methods 2.1 (single retrospective cohort, no separate evaluation dataset)
	5b	D;E	Specify the dates of collected participant data, including start/end of accrual and end of follow-up	Methods 2.1 (ethical approval 2019; IHC staining 2019-2022)
**Participants**	6a	D;E	Specify key elements of study setting, number and location of centres	Methods 2.1 (single tertiary referral center, Evangelismos Hospital)

Section/Topic	Item	D/E	Checklist item	Reported on page
	6b	D;E	Describe the eligibility criteria for study participants	Methods 2.1
	6c	D;E	Give details of any treatments received and how handled during development	Not applicable; staging/risk prediction model, not treatment-response model
**Data preparation**	7	D;E	Describe data pre-processing and quality checking, including across sociodemographic groups	Methods 2.2 (H-score scoring protocol); sociodemographic stratification not performed
**Outcome**	8a	D;E	Clearly define the outcome predicted and time horizon, rationale, consistency of assessment	Methods 2.1 (Masaoka-Koga stage, WHO risk group; cross-sectional outcome at diagnosis)
	8b	D;E	If outcome requires subjective interpretation, describe qualifications of assessors	Methods 2.1 (staging by treating surgical/pathology team per standard protocol)
	8c	D;E	Report any actions to blind assessment of the outcome	Not formally blinded; retrospective chart-based staging (Limitations)
**Predictors**	9a	D	Describe choice of initial predictors and any pre-selection before model building	Methods 2.2-2.3 (19 IHC H-score biomarkers selected based on prior biological rationale, refs 9-12)
	9b	D;E	Clearly define all predictors, including how/when measured	Methods 2.2 (H-score 0-300 scale, IHC protocol described)
	9c	D;E	If predictor measurement requires subjective interpretation, describe assessor qualifications	Methods 2.2 (H-score scored by trained pathologist per standardized protocol)
**Sample size**	10	D;E	Explain how study size was arrived at and justify sufficiency; sample size calculation details	Methods 2.1 and Limitations (retrospective cohort of available cases; no a priori sample size

Section/Topic	Item	D/E	Checklist item	Reported on page
				calculation; EPV reported as 7.3 Masaoka-Koga, 11.0 WHO)
**Missing data**	11	D;E	Describe how missing data were handled; reasons for omitting data	Methods 2.2 (complete-case analysis per biomarker combination; reasons = technical IHC failure/insufficient tissue)
**Analytical methods**	12a	D	Describe how data were used, including partitioning, considering sample size requirements	Methods 2.5 (100 independent stratified 70/30 train/test splits)
	12b	D	Describe how predictors were handled (functional form, rescaling, transformation, standardisation)	Methods 2.4 (continuous H-score values used directly; no transformation)
	12c	D	Specify model type, rationale, all model-building steps including hyperparameter tuning, internal validation method	Methods 2.3-2.5 (LR and XGBoost; XGBoost hyperparameters specified; nested feature selection within each split)
	12d	D;E	Describe handling of heterogeneity across clusters (e.g., hospitals)	Not applicable; single-center study
	12e	D;E	Specify all measures/plots used to evaluate performance (discrimination, calibration, clinical utility)	Methods 2.5-2.6 (AUC, recall, specificity, Brier score, ROC curves, 95% CI)
	12f	E	Describe any model updating arising from evaluation	Not applicable; no external evaluation/updating performed
	12g	E	Describe how model predictions were calculated for evaluation	Not applicable; internal evaluation only
**Class imbalance**	13	D;E	If class imbalance methods used, state why/how, and any recalibration	Methods 2.4 (SMOTE applied to Masaoka-Koga training folds

Section/Topic	Item	D/E	Checklist item	Reported on page
				only, due to 2.7:1 imbalance; not applied to balanced WHO cohort)
**Fairness**	14	D;E	Describe approaches used to address model fairness	Not assessed; single-center cohort without sociodemographic subgroup analysis (Limitations)
**Model output**	15	D	Specify model output and rationale/thresholds for classification	Methods 2.4 (predicted probability via logistic regression; default 0.5 threshold for binary classification)
**Training vs evaluation**	16	D;E	Identify differences between development and evaluation data	Not applicable; single cohort used throughout, no separate evaluation dataset (Limitations: no external validation)
**Ethical approval**	17	D;E	Name IRB/ethics committee and describe consent or waiver	Methods 2.1 (Evangelismos Hospital Ethics Committee approval, 2019)
**OPEN SCIENCE**				
**Funding**	18a	D;E	Give source of funding and role of funders	Funding statement (no external funding / self-funded doctoral research, as applicable)
Conflicts of interest	18b	D;E	Declare conflicts of interest and financial disclosures	Conflicts of Interest statement (none declared)
Protocol	18c	D;E	Indicate where study protocol can be accessed or state none prepared	State: no separate protocol was prepared prior to the study
Registration	18d	D;E	Provide registration information or state not registered	State: not registered (retrospective observational analysis)

Section/Topic	Item	D/E	Checklist item	Reported on page
Data sharing	18e	D;E	Provide details of data availability	Data Availability statement (H-score data available upon reasonable request, subject to ethical approval)
Code sharing	18f	D;E	Provide details of analytical code availability	Data Availability statement (Jupyter notebooks deposited on GitHub: github.com/lufakos83-cpu/TET-ML-Analysis)
**PATIENT & PUBLIC INVOLVEMENT**				
**Patient & Public Involvement**	19	D;E	Provide details of patient/public involvement or state no involvement	State: no patient or public involvement in study design (retrospective archival analysis)
**RESULTS**				
**Participants**	20a	D;E	Describe flow of participants, with/without outcome; diagram may help	Results 3.1 (n=81 Masaoka-Koga, n=89 WHO); flow diagram not included (Limitations)
	20b	D;E	Report characteristics overall and per data source, including key predictors, sample size, outcome events, missing data	Table 1 (Masaoka-Koga demographics), Table 2 (WHO demographics)
	20c	E	For model evaluation, compare distribution of predictors with development data	Not applicable; no separate evaluation dataset
**Model development**	21	D;E	Specify number of participants and outcome events in each analysis	Tables 5-12 (N reported per biomarker combination, reflecting complete-case analysis); new EPV table in Results 3.3

Section/Topic	Item	D/E	Checklist item	Reported on page
**Model specification**	22	D	Provide full prediction model details to allow third-party implementation	Table 14 (LR coefficients for both optimal models reported in full)
**Model performance**	23a	D;E	Report performance estimates with confidence intervals, including subgroups	Tables 5-13 (AUC, recall, specificity, Brier score, 95% CI for all models); Figures 2-3 (ROC curves)
	23b	D;E	Report heterogeneity in performance across clusters, if examined	Not applicable; single-center cohort
**Model updating**	24	E	Report results of any model updating	Not applicable; no external evaluation performed
**DISCUSSION**				
**Interpretation**	25	D;E	Give overall interpretation of main results, including fairness considerations	Discussion, opening paragraphs
**Limitations**	26	D;E	Discuss limitations (sample size, overfitting, missing data) and effects on bias, uncertainty, generalizability	Limitations section (EPV, absence of external validation, complete-case analysis, single-center design, self-citation of IHC protocol references, sociodemographic non-assessment)
**Usability of the model**	27a	D	Describe how poor quality/unavailable input data should be assessed/handled	Discussion (Clinical implications subsection): recommend standardized IHC protocol and pathologist training before implementation
	27b	D	Specify required user interaction and level of expertise required	Discussion: requires trained pathologist for H-score scoring; no specialized computational

Section/Topic	Item	D/E	Checklist item	Reported on page
				expertise required for score calculation
	27c	D;E	Discuss next steps for future research re: applicability/generalizability	Discussion, Future Directions: multicenter external validation, prospective cohort, calibration assessment

Note: This study reports model development only; no independent external evaluation dataset was used. Items relating exclusively to model evaluation (E) on a separate dataset, including 12f, 12g, 20c, and 24, are marked 'Not applicable' accordingly. Page numbers should be verified against final manuscript pagination prior to submission.

Supplementary Figure S1. Completed TRIPOD+AI checklist for the present study, reporting compliance with the TRIPOD+AI guidelines for studies developing or evaluating multivariable prediction models using artificial intelligence and machine learning methods. D, items relevant to model development; E, items relevant to model evaluation; D;E, items applicable to both. Items not applicable to this single-center development study without external evaluation are indicated accordingly.
