# Supplementary material for: Machine Learning-Based Prediction of Masaoka–Koga Stage and WHO Histological Risk Group in Thymic Epithelial Tumors Using Biomarker Combinations
Source: Diagnostics (Basel). 2026 Jul 7;16(13):2118. doi: 10.3390/diagnostics16132118 (PMC13360224; doi:10.3390/diagnostics16132118)
Supplement: Supplementary file 1 [file diagnostics-16-02118-s001.zip › Supplementary Figure S2.pdf]

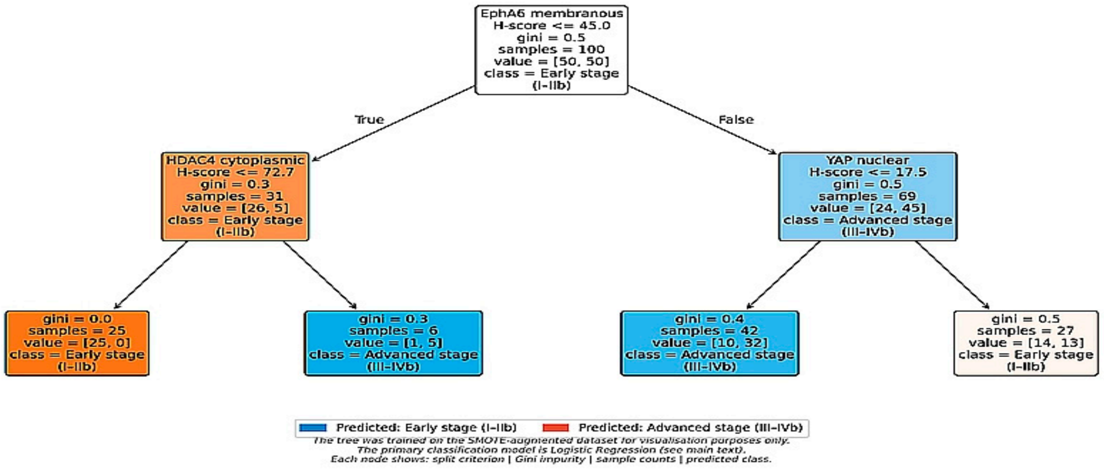

Supplementary Figure S2. Exploratory Decision Tree (depth = 2) for the optimal Masaoka-Koga trivariate model (EphA6 membranous + YAP nuclear + HDAC4 cytoplasmic; trained on SMOTE-augmented dataset). Primary split: EphA6 membranous ≤ 45. For exploratory visualization only; primary model is Logistic Regression.
