# Supplementary material for: Machine Learning-Based Prediction of Masaoka–Koga Stage and WHO Histological Risk Group in Thymic Epithelial Tumors Using Biomarker Combinations
Source: Diagnostics (Basel). 2026 Jul 7;16(13):2118. doi: 10.3390/diagnostics16132118 (PMC13360224; doi:10.3390/diagnostics16132118)
Supplement: Supplementary file 1 [file diagnostics-16-02118-s001.zip › Supplementary Figure S3.pdf]

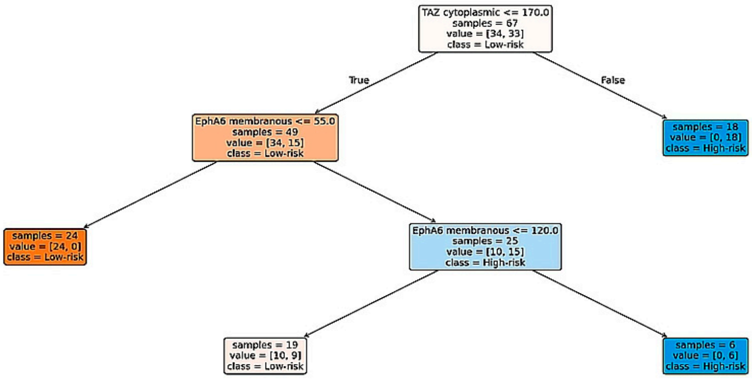

Supplementary Figure S3. Exploratory Decision Tree (depth = 3) for the optimal WHO trivariate model (TAZ cytoplasmic + EphA6 membranous + YAP nuclear; n = 67). Two high-purity leaf nodes: TAZ cytoplasmic > 170 → High-risk (18/18); TAZ ≤ 170 and EphA6 membranous ≤ 55 → Low-risk (24/24). For exploratory visualization only; primary model is Logistic Regression.
