# Supplementary material for: Machine Learning-Based Prediction of Masaoka–Koga Stage and WHO Histological Risk Group in Thymic Epithelial Tumors Using Biomarker Combinations
Source: Diagnostics (Basel). 2026 Jul 7;16(13):2118. doi: 10.3390/diagnostics16132118 (PMC13360224; doi:10.3390/diagnostics16132118)
Supplement: Supplementary file 1 [file diagnostics-16-02118-s001.zip › Supplementary Figure S4.pdf]

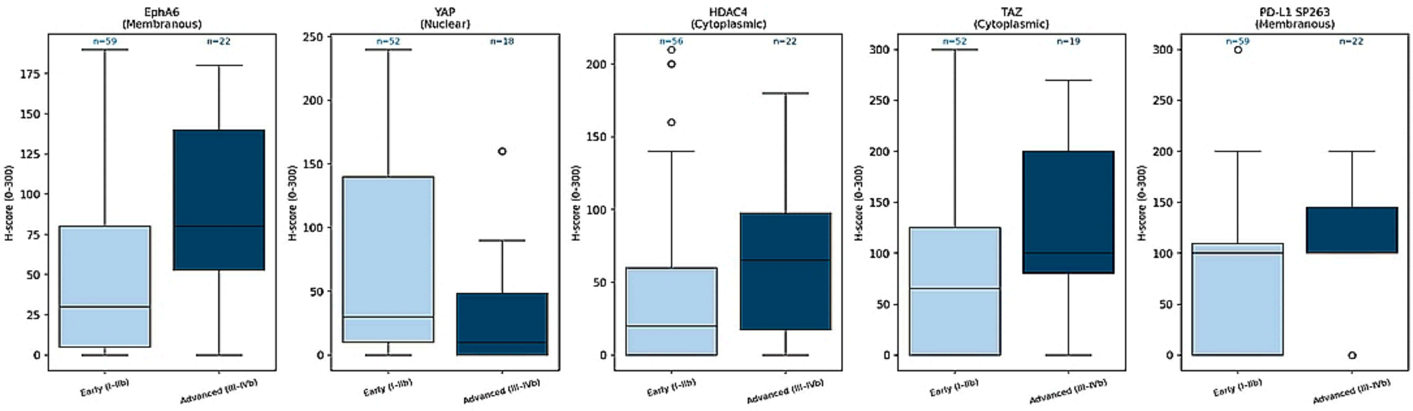

Supplementary Figure S4. H-score distributions for the five top-performing Masaoka-Koga biomarkers (EphA6 membranous, YAP nuclear, HDAC4 cytoplasmic, TAZ cytoplasmic, PD-L1 SP263) stratified by Masaoka-Koga stage group (early I–IIb vs advanced III–IVb). EphA6, HDAC4, and TAZ are elevated in advanced-stage tumors; YAP shows the opposite pattern. Box plots show median and IQR.
