# Supplementary material for: Machine Learning-Based Prediction of Masaoka–Koga Stage and WHO Histological Risk Group in Thymic Epithelial Tumors Using Biomarker Combinations
Source: Diagnostics (Basel). 2026 Jul 7;16(13):2118. doi: 10.3390/diagnostics16132118 (PMC13360224; doi:10.3390/diagnostics16132118)
Supplement: Supplementary file 1 [file diagnostics-16-02118-s001.zip › Supplementary Figure S5.pdf]

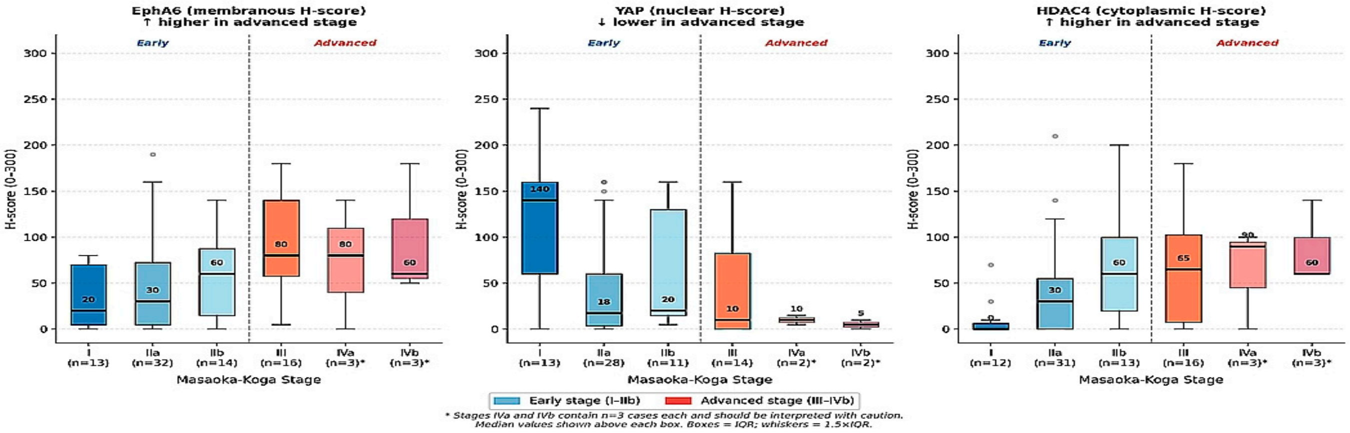

Supplementary Figure S5. H-score distributions for the optimal Masaoka-Koga trivariate markers (EphA6 membranous, YAP nuclear, HDAC4 cytoplasmic) stratified across all six individual Masaoka-Koga stages (I, IIa, IIb, III, IVa, IVb). A progressive directional gradient is visible for all three markers. Stages IVa and IVb contain n = 3 cases each and should be interpreted with caution.
