# Supplementary material for: Machine Learning-Based Prediction of Masaoka–Koga Stage and WHO Histological Risk Group in Thymic Epithelial Tumors Using Biomarker Combinations
Source: Diagnostics (Basel). 2026 Jul 7;16(13):2118. doi: 10.3390/diagnostics16132118 (PMC13360224; doi:10.3390/diagnostics16132118)
Supplement: Supplementary file 1 [file diagnostics-16-02118-s001.zip › Supplementary Figure S6.pdf]

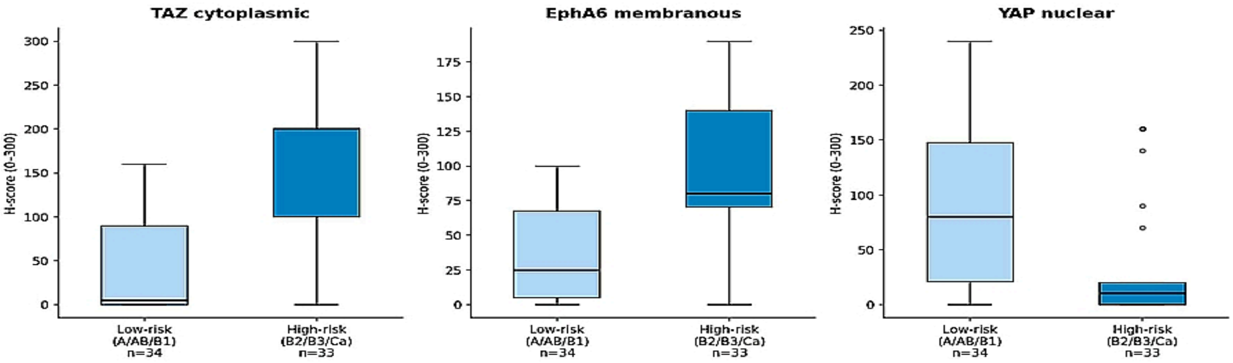

Supplementary Figure S6. H-score distributions for the optimal WHO trivariate markers (TAZ cytoplasmic, EphA6 membranous, YAP nuclear) stratified by WHO histological risk group (low-risk: A/AB/B1, n = 34; high-risk: B2/B3/carcinoma, n = 33).
