# Supplementary material for: Machine Learning-Based Prediction of Masaoka–Koga Stage and WHO Histological Risk Group in Thymic Epithelial Tumors Using Biomarker Combinations
Source: Diagnostics (Basel). 2026 Jul 7;16(13):2118. doi: 10.3390/diagnostics16132118 (PMC13360224; doi:10.3390/diagnostics16132118)
Supplement: Supplementary file 1 [file diagnostics-16-02118-s001.zip › Supplementary Figure S7.pdf]

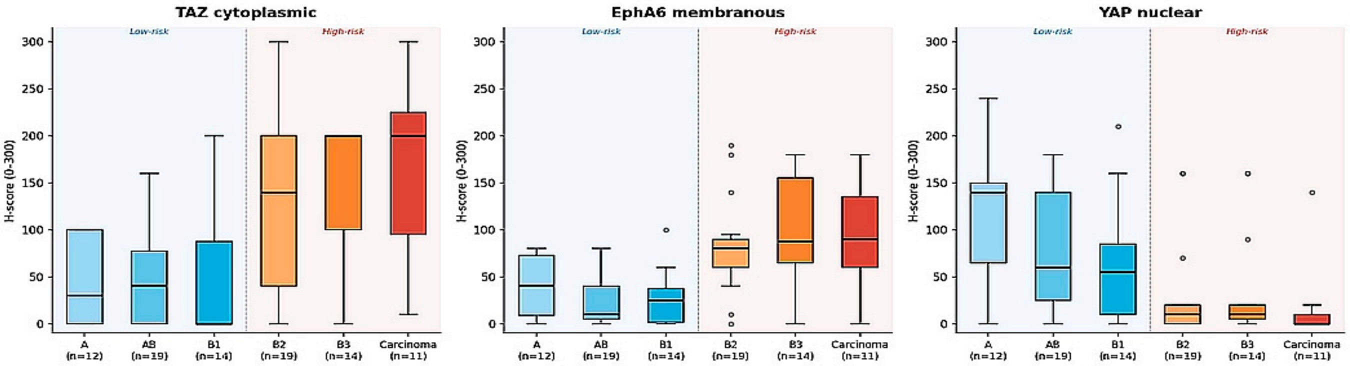

Supplementary Figure S7. H-score distributions for the optimal WHO trivariate markers stratified by WHO histological subtype (A → AB → B1 → B2 → B3 → Carcinoma). A progressive directional gradient is visible for all three markers. Micronodular thymoma (n = 2) excluded.
