# Supplementary material for: Machine Learning-Based Prediction of Masaoka–Koga Stage and WHO Histological Risk Group in Thymic Epithelial Tumors Using Biomarker Combinations
Source: Diagnostics (Basel). 2026 Jul 7;16(13):2118. doi: 10.3390/diagnostics16132118 (PMC13360224; doi:10.3390/diagnostics16132118)
Supplement: Supplementary file 1 [file diagnostics-16-02118-s001.zip › Supplementary Figure S8.pdf]

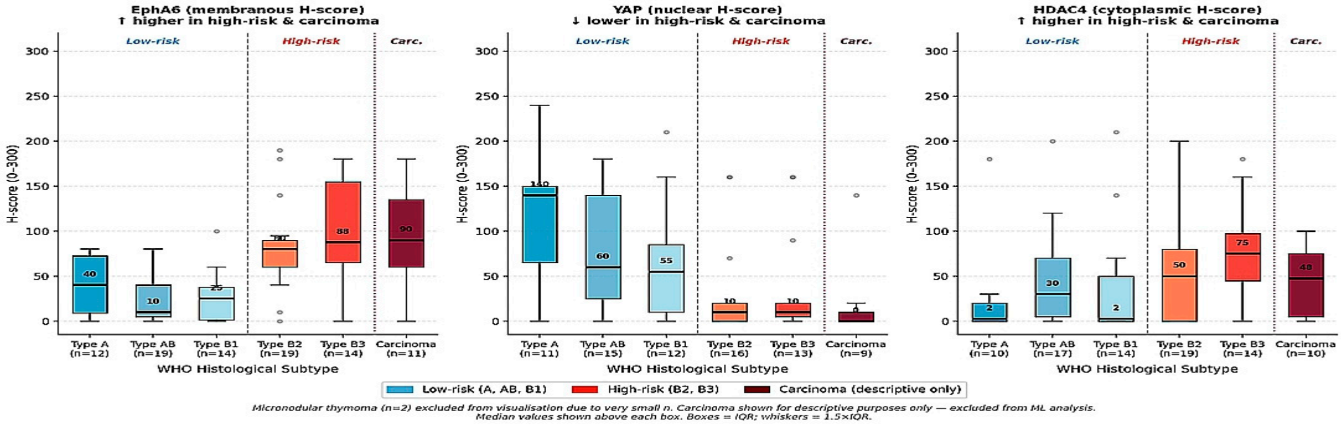

Supplementary Figure S8. H-score distributions for the optimal Masaoka-Koga trivariate markers (EphA6 membranous, YAP nuclear, HDAC4 cytoplasmic) stratified by WHO histological subtype, illustrating the cross-endpoint biological gradient. EphA6 and HDAC4 increase progressively from low-risk to high-risk subtypes; YAP decreases. Micronodular thymoma (n = 2) excluded.
